# Improving Health-Related Quality of Life of Patients With an Ostomy Using a Novel Digital Wearable Device: Protocol for a Pilot Study

**DOI:** 10.2196/resprot.7470

**Published:** 2018-03-26

**Authors:** Dara Rouholiman, Jamison G Gamble, Sylvie D Dobrota, Ellen M Encisco, Ashish G Shah, Francisco J Grajales III, Larry F Chu

**Affiliations:** ^1^ Stanford Medicine X Digital Health Laboratory Medicine X Program, Department of Anesthesiology, Perioperative and Pain Medicine Stanford University School of Medicine Stanford, CA United States; ^2^ Medical College of Georgia Augusta University Augusta, GA United States; ^3^ Centre for Social Innovation & Impact Investing Sauder School of Business University of British Columbia Vancouver, BC Canada

**Keywords:** ostomy, quality of life, eHealth

## Abstract

**Background:**

Ostomy surgeries involving the placement of an ostomy bag (eg, colostomy, ileostomy, urostomy, etc) have been shown to have a negative impact on health-related quality of life. To date, no studies have been conducted examining what impact, if any, wearable biosensors have on the health-related quality of life of ostomy patients.

**Objective:**

In the present study, we plan to assess the quality of life of ostomy patients using the Ostom-i alert sensor, a portable, wearable, Bluetooth-linked biosensor that facilitates easier ostomy bag output measurements. We hypothesize that using the Ostom-i alert sensor will result in an improved, ostomy-specific, health-related quality of life as compared to baseline measurement before the use of the sensor.

**Methods:**

A total of 20 ostomy patients will be screened and recruited to participate in this prospective, observational, cross-over pilot study using an Ostom-i alert sensor for one month. The primary outcome of this study will compare ostomy-specific, health-related quality of life at baseline (prior to Ostom-i alert sensor use) to ostomy-specific, health-related quality of life after 2 and 4 weeks of Ostom-i use by utilizing the City of Hope Quality of Life Questionnaire for Patients with an Ostomy. Secondary outcomes of general health-related quality of life and adjustment to ostomy will be evaluated using the Medical Outcomes Study 36-item short form health survey and the Olbrisch Ostomy Adjustment Scale Short Form 2.

**Results:**

The project was funded by the Department of Anesthesiology, Perioperative and Pain Medicine at Stanford University School of Medicine. Enrollment is currently underway and data analysis is expected to be completed in 2018.

**Conclusions:**

Proposed benefits of mobile, internet-linked personal health monitors, such as the Ostom-i, include a reduction in the cost of care by reducing resource utilization and infection rates, improving patient-provider communication, reducing time spent as an inpatient as well as improved quality of life. Prior studies have demonstrated decreased health-related quality of life in patients with an ostomy bag. We aim to examine the extent to which the Ostom-i alert sensor affects the health-related quality of life of its users. The Ostom-i alert sensor has the potential to improve quality of life of users by giving them the freedom and confidence to partake in daily activities with the knowledge that they can check how full their ostomy bag is in a private, discrete manner.

**Trial Registration:**

ClinicalTrials.gov NCT02319434; https://clinicaltrials.gov/ct2/show/NCT02319434 (Archived at WebCite at http://www.webcitation.org/6xhFDThmq)

## Introduction

### Background and Rationale

Ostomy surgeries such as colostomy (large bowel), ileostomy (small bowel), and urostomy (bladder), which require the use of an ostomy bag either temporarily or permanently, may result in a change in health-related quality of life as patients adjust to life with their ostomy [1-4]. While the average wear time of an ostomy bag in the United States has been reported to be 4.8 days, up to 40%-60% of stoma will never be reversed and many patients with severe inflammatory bowel disease or advanced colorectal cancer may wear an ostomy bag long-term [5-7]. Colostomies requiring an ostomy bag are common in patients with colorectal cancer, which as of 2016 was the second most commonly diagnosed cancer in men and the third most commonly diagnosed cancer in women in the United States [8]. In 2016, the number of newly diagnosed cases of colorectal cancer was 724,690 and 727,350 in men and women, respectively. Estimates suggest that there will be 910,190 newly diagnosed cases of colorectal cancer in men and 885,940 new cases in women in 2026. It has been reported that colostomy surgery is more common in patients with rectal cancer (29%) than for patients with colon cancer (12%) [8].

While ostomy surgery may improve health-related quality of life by reducing disease burden, it can often decrease general quality of life in other ways. Common themes in health-related quality of life for ostomy patients include factors such as social adjustment, fatigue, pain, leakage, physical functioning, changes in clothing, and diet [9]. A significant concern of patients with an ostomy bag is return to work, work efficiency, and worries about social and personal life due to the presence of the ostomy bag [10]. While factors such as coping, acceptance, and availability of ostomy specialist to patients have been identified as methods to improve health-related quality of life of ostomy patients, there have been few technological advancements geared towards improving health-related quality of life of individuals with an ostomy bag [11,12]. Existing portable technologies are primarily focused on the cleaning of the ostomy bag, such as a 2004 patent allowing the user to clean the bag more completely and with greater ease; however, no mobile health (mHealth) technologies currently exist to alert the wearer as to the fullness of their ostomy bag [13].

We are conducting a prospective trial to evaluate the impact of the Ostom-i alert sensor on short-term, health-related quality of life of ostomy patients. The Ostom-i alert sensor is a wearable device intended to make life easier for patients with ostomy bags by allowing for easier output measurements and anticipation of bag changes via a Bluetooth connection to their mobile smart phone. Using the City of Hope Quality of Life Questionnaire for a Patient with an Ostomy (CoH-QOL-Ostomy), we determine to what extent, if any, the Ostom-i sensor affects health-related quality of life of the user [14].

### Objective

We hypothesize that using the Ostom-i alert sensor will result in an improved ostomy-specific, health-related quality of life as compared to baseline measurement before the use of the sensor. We intend to assess the change in ostomy-specific health-related quality of life, with the Ostom-i alert sensor. We will use the City of Hope Quality of Life Questionnaire for Patients with an Ostomy. Secondarily, we aim to measure the change in general health-related quality of life and ostomy adjustment using the Medical Outcomes Study 36-item short form health survey (SF-36) and the Olbrisch Ostomy Adjustment Scale Short Form 2 (OAS-SF2), respectively [15,16].

## Methods

### Participants, Interventions, and Outcomes

#### Study Setting

Patient recruitment will occur at the Stanford University Medical Center. Data analysis and all other matters related to manuscript drafting will occur at the Stanford University School of Medicine. Both settings are located in Palo Alto, California within Santa Clara County.

#### Eligibility Criteria

Recruited patients will be required to meet the eligibility criteria outlined in Textbox 1. Any participants who do not meet our inclusion criteria will be excluded from the study. Our decision to exclude participants who have had an ostomy for less than 6 months was based off the work of Husain and Cataldo [6], who determined that 93% of ostomy-related complications occur within the first six months after ostomy surgery. Furthermore, they determined that psychological adjustment to the ostomy occurs 6 to 10 weeks after surgery, implying that participants in our population will be fully psychologically adjusted to their ostomy [6]. We are limiting our study to patients with colostomy, ileostomy or urostomy. Large urostomy bags will not work with the Ostom-i sensor and thus individuals with large urostomy bags (>9 cm) will be excluded from the study (see Textbox 1 for a complete list of patient inclusion and exclusion criteria).

#### Recruitment

Participants will be recruited from the Stanford University Medical Center via word of mouth, online advertisements, flyers posted in the hospital as well as referrals from ostomy physicians and nursing staff. Patient recruitment will be facilitated with the help of a number of ostomy nurses at Stanford. Persons interested in the study will be directed to a Web page which includes information about the study sensor, what study participation involves, and a link to a complete online eligibility survey. Participants will not receive monetary compensation for participating in this study, but will be able to keep the Ostom-i sensor which retails for US $125.

**Study eligibility inclusion and exclusion criteria.**

**Inclusion Criteria**

- Ability to read and understand English
- 18-80 years of age
- Current use of an ostomy bag
- Use of an ostomy bag for 6 months or more
- Use of an ostomy bag for the duration of the study
- Access to and ability to use an iOS or Android smartphone, iPod Touch or tablet

**Exclusion Criteria**

- Ostomy bag other than colostomy, ileostomy or urostomy
- Urostomy bag larger than 9 cm

#### Screening

Participant screening will occur either online via the Web page, in person, or over the phone. The online screening survey will use Stanford Medicine Qualtrics to collect and analyze data on eligible persons [17].

#### Randomization

Patients will serve in both the control and interventional arms of this cross-over pilot study. A cross-over design is advantageous for this pilot study as it allows patients to serve as their own control, therefore variances attributable to confounding factors [18,19]. Once eligibility is confirmed, consent will be obtained and the baseline survey will be given (CoH-QOL-Ostomy, SF-36 and OAS-SF2). After completion of the baseline survey, the participant will be given their Ostom-i alert sensor along with a video tutorial which explains how to use the device.

#### Intervention

**Figure 1:**
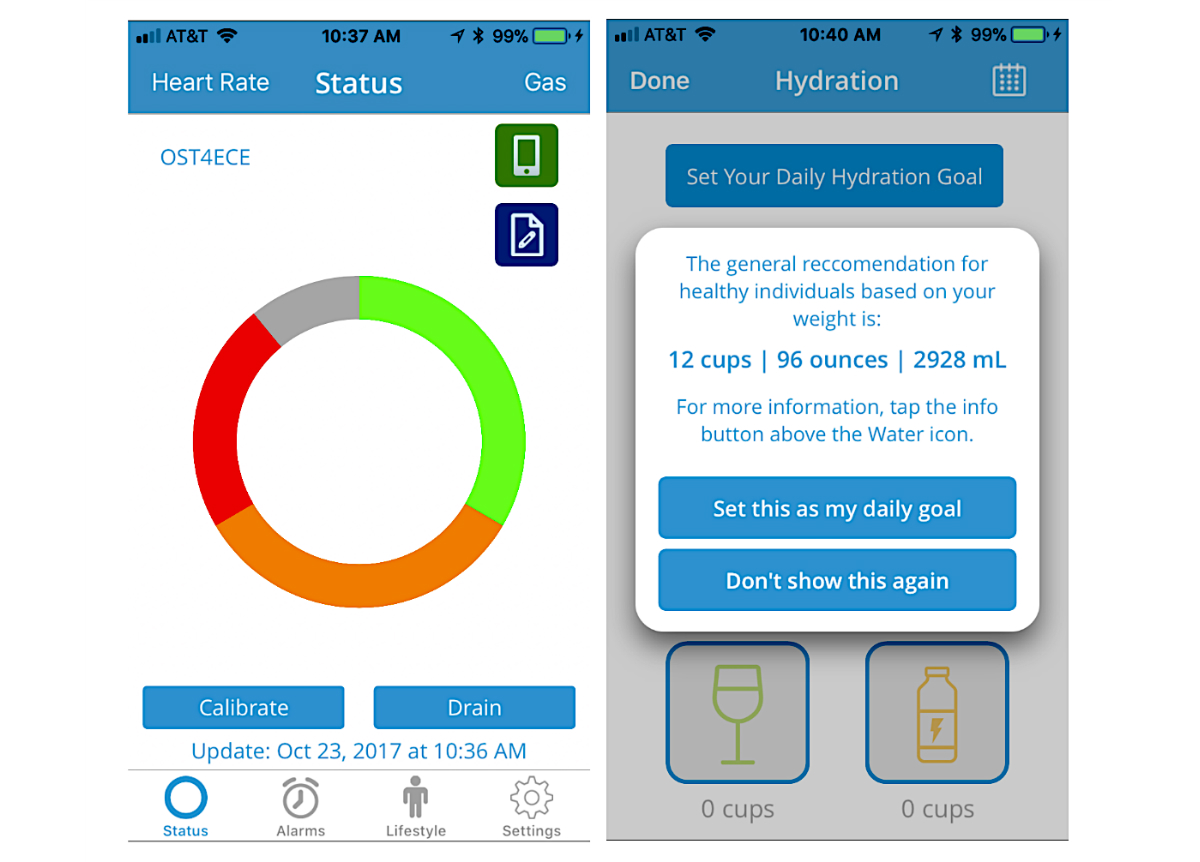
Screenshots from the Ostom-i patient app showing status, hydration, and graph.

**Figure 2:**
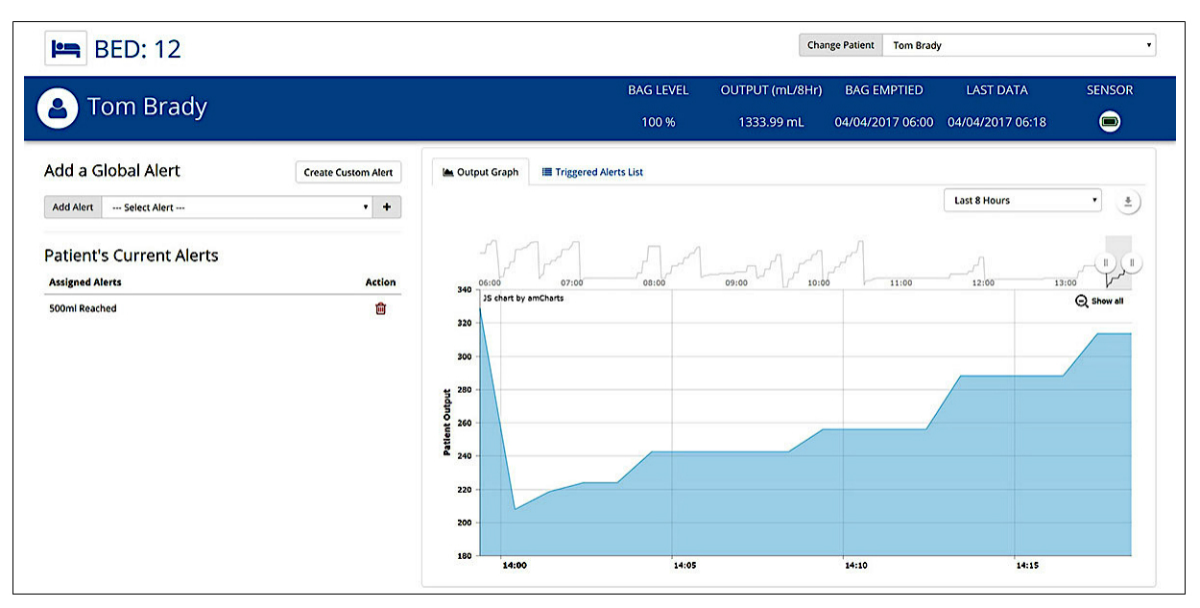
Screenshot from the Ostom-i patient app showing user interface, status, hydration, and graph.

Once completing the baseline survey, patients will enter the intervention arm of the study where they will receive an Ostom-i sensor which they will use over the course of 4 weeks. Two and 4 weeks after receiving the sensor, primary and secondary outcome measures will be assessed. The Ostom-i is a flexible, Bluetooth-linked sensor that attaches to the patient’s ostomy bag. The sensor portion of the Ostom-i device is a flexible potentiometer produced by Spectra Symbol [20]. The sensor determines the level of the ostomy bag based on the angle of flex it experiences and automatically adjusts when the user is laying down or standing up. The sensor can be adjusted in size from 7 cm to 9 cm to fit a variety of ostomy bag sizes. Data collected by the sensor is sent via Bluetooth to the user’s iOS or Android device and provides alerts informing the wearer of the level of their ostomy bag (Figure 1 and 2).

#### Participant Timeline

**Figure 3:**
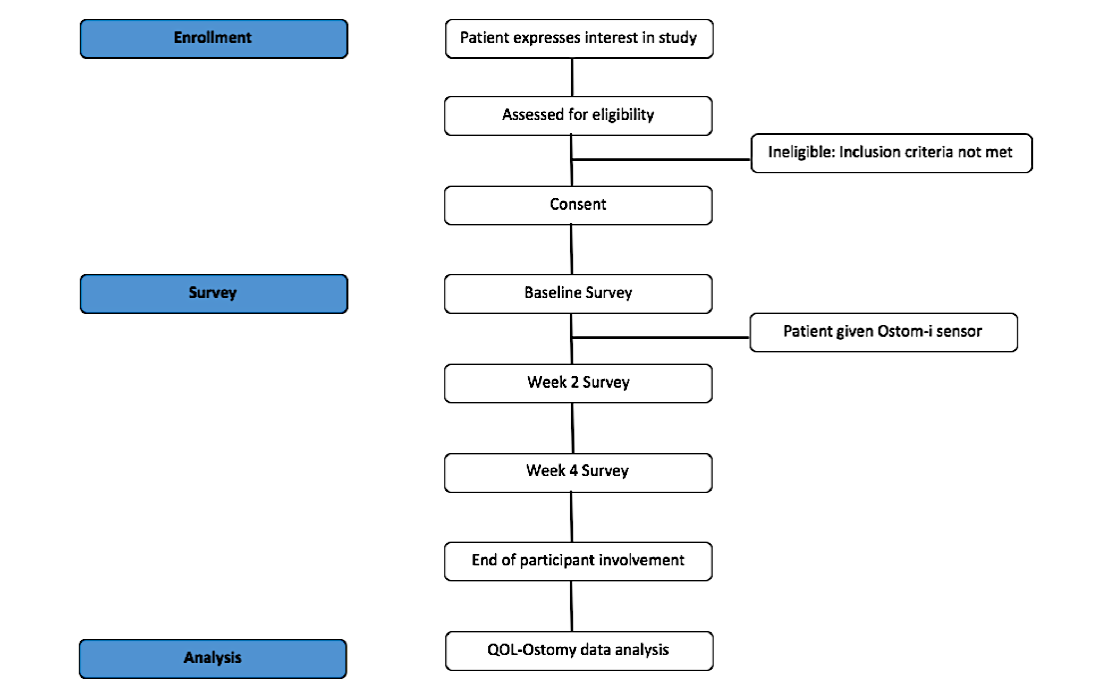
Participant flowchart. QOL-Ostomy: Quality of Life Questionnaire for a Patient with an Ostomy.

Participants will retake all 3 surveys after 2 and 4 weeks of device use as previous validation studies of these surveys have used 2-4 week intervals [16,21]. Following completion of the week 4, survey participant involvement in the study will end (see Figure 3 for the study flowchart). Participants will have the option to take the week 2 and week 4 surveys either in person at the hospital or at home, using a paper-based or online format.

#### Primary Outcome Measures

The modified CoH-QOL-Ostomy is an ostomy-specific, health-related quality of life instrument with four dimensions. The four dimensions—physical, psychological, social, and spiritual well-being are defined in Table 1. Ostomy-specific, health-related quality of life is calculated by summing scores for each question then dividing by the total number of questions (ie, 43 questions). Total scores for each of the four dimensions are calculated by adding scores on all dimension items and dividing by total number of dimension items. Ostomy-specific, health-related quality of life will be measured at baseline prior to receiving the Ostom-i device, then again after 2 and 4 weeks of device use.

#### Secondary Outcome Measures

Two secondary outcome measures will be utilized including the SF-36 and the OAS-SF2. The SF-36 is a commonly used general health-related quality of life instrument. In this study, it will be used to compare ostomy-specific, health related quality of life to general (nonostomy-specific) health-related quality of life. The OAS-SF2 is used to examine subjective response to ostomy as well as psychological adjustment to the ostomy.

#### Null Hypothesis and Sample Size

Our null hypothesis states that there will be no improvement in ostomy-specific, health-related quality of life as measured by the CoH-QOL-Ostomy, compared to baseline measurements prior to sensor use. To calculate the desired sample size to test our null hypothesis, the following paired *t*-test formula was used:


*n=[σ_d_^2^(Z_power_+Z_α/2_)^2^]/μ_d_^2^*

where our mean and variance is based on the work of Gemmill et al [22], who examined 307 ostomy patients and reported a mean of 8.0 (SD 1.7) for the social well-being dimension within the CoH-QOL-Ostomy, with 80% power and 95% confidence [22]. Our sample size calculation (n=16 for a 15% difference in the dimension of social well-being) was again based on Gemmill et al [22], who reported social well-being to have a higher mean (8.0) than overall quality of life (mean 7.7).

**Table 1:** City of Hope quality of life dimension definitions obtained from Gemmill et al [22].

Dimension	Definition
Physical well-being	Physical symptoms and functional ability
Psychological well-being	Emotional components of the illness including positive as well as negative aspects
Social well-being	Role of the patient with the family and society including occupational, sexual, and personal relationships
Spiritual well-being	Religious aspects and existential concerns

**Table 2:** Desired sample size number (N) based on % difference.

% Difference	Absolute change in dimension of well-being (μd)	Sample size (N)
10	0.8	35
15	1.2	16
30	2.4	4
40	3.2	2

Furthermore, Gemmill et al [22] report a lower SD for the dimension of social well-being (SD 1.7) as compared to physical well-being (SD 1.8), psychological well-being (SD 1.9), and spiritual well-being (SD 2.3). Thus, we chose to base our sample size calculation on the dimension of social well-being. With a sample size calculation of n=4 for 30% difference, we are concerned that our results would lack generalizability. Therefore, by increasing our sample size to 20, we hope that our results will be more generalizable and may help account for a potential 20% attrition rate during recruitment (Table 2).

### Trial Design

The design of our pilot study is a prospective, single group, observational, prepost cross-over trial. Ethical approval was obtained from the Institutional Review Board at Stanford University (Protocol #32211). This study is registered at ClinicalTrials.gov (NCT02319434).

### Data Collection, Management, and Analysis

#### Primary Outcome Data Collection Methods

Our primary outcome, change in ostomy-specific, health-related quality of life from baseline, will be measured using the modified CoH-QOL-Ostomy. This survey was designed and studied for reliability and validity by Grant et al [14] with an overall questionnaire alpha of .95, suggesting strong consistency. The survey can be divided into 6 sections: 1) social adjustment to ostomy (coefficient alpha=.90, correlation to single quality of life item: *r*=.44, *P*>.001); 2) general quality of psychological well-being (coefficient alpha=.83, correlation to single quality of life item: *r*=.76, *P*>.001); 3) general quality of physical well-being (coefficient alpha=.88, correlation to single quality of life item: *r*=.39, *P*>.001); 4) disease-specific effects on physical well-being (coefficient alpha=.77, correlation to single quality of life item: *r*=.24, *P*>.001); 5) general quality of spiritual well-being (coefficient alpha=.81, correlation to single quality of life item: *r*=.51, *P*>.001); and 6) disease specific effects on psychological well-being (coefficient alpha=.82, correlation to single quality of life item: *r*=.38, *P*>.001) [14]. This validated survey has successfully been used by number of studies examining ostomy-related quality of life [22-27].

#### Secondary Outcome Data Collection Methods

Our secondary outcomes, general health-related quality of life and psychological adjustment to the ostomy, will be measured using the SF-36 and the OAS-SF2. The SF-36 involves a scale which measures 8 health profiles including physical functioning (PF), role limitations due to physical health problems (RP), bodily pain (BP), general health perceptions (GH), vitality (VT), social functioning (SF), role limitations due to emotional problems (RE), and mental health (MH). The SF-36 also yields physical and mental health summary measures and is scored using a Likert scale [28]. Furthermore, the SF-36 has been validated and was found to be reliable across a diverse group of patients with various physical and psychological issues [28,29]. Each of the 8 dimensions of the SF-36 have been found to have a Cronbach alpha statistic greater than the minimum standard of .70; PF (alpha=.90), SF (alpha=.76), RP (alpha=.88), RE alpha=.80), MH (alpha=.83), VT (alpha=.85), GH (alpha=.78), and BP (alpha=.82) suggesting strong internal-consistency and reliability [28,30,31].

Adjustment to ostomy will be evaluated using OAS-SF2 [16]. The OAS is a subjective scale specific to persons with an ostomy, and examines social, psychological, and sexual functioning adjustment to living with an ostomy. The OAS is measured on a 6-point Likert scale and contains 34 items. Cronbach’s alpha for the scale was calculated at alpha=.85 with a test-retest reliability at *r*=.72 and later confirmed in studies of Swedish, Norwegian, and Chinese patients with an ostomy [32-34]. Two, 17-question short forms (short form 1 and short form 2) were created by Olbrisch based on the original 34 questions. It was determined that each short form could be used independently without compromising reliability or validity of the 34-question-long form (*r*=.96). Furthermore, short form test-retest consistency and reliability was determined to be *r*=.69 [21].

#### Data Management

Survey assessments will be collected via Stanford Qualtrics (Qualtrics, Provo, UT) survey or pen-and-paper [17]. Data will be entered into the Stanford Research Electronic Data Capture (REDCap) databases (Vanderbilt, Nashville, TN) [35]. All data will be entered and de-identified by trained staff and undergo data quality and accuracy checks.

#### Statistical Analysis

Data will be presented as mean (SD). Changes between pre- and postintervention quality of life will be assessed using a dependent participant’s paired *t*-test with 95% confidence interval. Depending on participant retention throughout the course of the study, we may choose to use mixed model regression analysis which would allow us to incorporate incomplete data sets from participants who might not complete the study. Furthermore, we also may choose to use repeated measures analysis of variance to examine differences in population mean scores over the 3 study sessions.

### Monitoring

#### Data Monitoring

A data monitoring committee (DMC) will not be used in this study. In accordance with the United States Food and Drug Administration Title 21 (21 Code of Federal Regulation 812) and the Stanford University Institutional Review Board (IRB), the Ostom-i alert sensor was not deemed to pose a significant risk to study participants [36]. Furthermore, there is an overall low level of concern for patient safety with the Ostom-i alert sensor. Given the short timeframe of the study, a DMC may not be practical and it is not likely that a DMC will aid in improving the scientific validity of this study [37]. This study is in full compliance with the guidelines outlined by ClinicalTrials.gov.

#### Risk and Side Effects

Due to the minimal intervention in this study, participants are at very low risk for adverse events. Should any adverse events occur, they will be systematically logged and reported to ClinicalTrials.gov. Adverse events involving the ostomy site or the ostomy bag, which are not related to the Ostom-i alert sensor, will be directed to the study participant’s gastroenterologist.

#### Auditing

This study is being conducted independently from the Ostom-i alert sensor parent company, 11 Health and Technologies, LLC. 11 Health and Technologies, LLC will not audit any aspect of the study. Due to the short duration of this study (12 months), auditing is not deemed a necessary component of our protocol.

### Ethics and Dissemination

#### Research Ethics Approval and Protocol Amendments

Ethical approval was obtained from the IRB at Stanford University (Protocol #32211). Any amendments made to the study protocol will be immediately reported to the IRB at Stanford University as well as to ClinicalTrials.gov.

#### Consent or Assent and Confidentiality

Informed consent will be obtained from study participants by study research personnel prior to in-person baseline evaluation. Phone conversations and in-person visits will take place in a private room to protect patient privacy. Data collected by the Ostom-i alert sensor will remain on the participant’s personal device (iPhone, Android, tablet etc) for the duration of the study. Meetings and phone calls will not be recorded and will only involve necessary study staff. 

Data collected from participants will include demographic information such as names, telephone numbers, addresses, birthdates, email addresses, illness/diagnosis, gender, age, height, weight, ethnicity, marital status, records of waste output as measured by the Ostom-i alert sensor as well as results from the CoH-QOL-Ostomy, SF-36, and Olbrisch’s Ostomy Adjustment Scale. Demographic and survey data will be collected and stored in a secure database on an encrypted computer. 

Participants will be assigned a random, 2-digit numerical identifier which will be stored in a locked safe in the laboratory. Collected data will also be stored in the secure REDCap database and necessary data transfer will occur using secure methods (eg, emails marked as secure). All aspects of data security in this study are in full compliance with the Stanford University Office of Audit, Compliance, Risk and Privacy.

#### Access to Data

Final trial data will only be available to study research personnel. All necessary demographic and results data will be uploaded to ClinicalTrials.gov in accordance with their rules and regulations.

#### Ancillary and Posttrial Care

Should study staff identify health issues in participants over the course of the study, they will be immediately referred to their primary care physician or gastroenterologist. Furthermore, study physicians will be available to answer study participant questions. No poststudy follow-up of participants will occur.

#### Dissemination Policy

The study authors plan to publish collected data in a peer-reviewed journal (to be determined at a later date). Furthermore, this study is fully compliant with the guidelines set forth by ClinicalTrials.gov and as such all necessary information will be made available in a timely manner. All listed authors and/or contributors are compliant with guidelines outlined by the International Committee of Medical Journal Editors for author inclusion in a published work. Public access to the study protocol and other necessary aspects will be made available through our ClinicalTrials.gov identifier (NCT02319434).

## Results

The project was funded by the Department of Anesthesiology, Perioperative and Pain Medicine at Stanford University School of Medicine. Enrollment is currently underway and data analysis is expected to be completed in 2018.

## Discussion

The Ostom-i alert sensor is a novel, wearable sensor that allows for easier output measurements and anticipation of ostomy bag changes via Bluetooth connection to a mobile phone. The Ostom-i alert sensor has the potential to improve quality of life of users by giving them freedom and confidence to partake in daily activities with the knowledge that they can check how full their ostomy bag is in a private, discrete manner. To examine the extent to which the Ostom-i alert sensor affects quality of life, 20 participants will be recruited to wear the Ostom-i alert sensor for 1 month. Health-related quality of life will be determined by using the CoH-QOL-Ostomy. This survey will be given at baseline to individuals who have had an ostomy bag for 6 months or longer, then again 2 and 4 weeks after beginning with the Ostom-i sensor. Ultimately, we anticipate that the Ostom-i alert sensor may improve health-related quality of life as measured by the CoH-QOL-Ostomy.

Proposed benefits of mHealth technologies, such as the Ostom-i, include a reduction in the cost of care by lowering resource utilization and infection rates, improving patient-provider communication, and reducing time spent as an inpatient [38-40].

A number of mHealth technologies, such as the Ostom-i alert sensor have recently been released including devices, such as the Withings Blood Pressure Monitor, the Sanofi iBGStar Blood Glucose Meter, and the AliveCor Mobile ECG. While these devices have all been validated in the peer-reviewed literature, few studies have examined to what extent, if any, they reduce burden on health care systems [41-43]. 

A 2006 study by Leijdekkers and Gay [44] analyzed a novel, cell phone–linked heart monitor and suggest that by visualizing their personal cardiac data in real time, users are less likely to visit the hospital, which in turn reduces hospital staff workload, reduces costs of patient-provider communication, and improves patient self-care [44]. Free et al [45] examined the literature and found 42 controlled trials of mobile technology–based systems aimed at improving health care service delivery [45]. They report only a modest benefit towards clinical management and diagnosis with the use of mobile technologies. 

Bloss et al [46] examined the extent to which the Withings Blood Pressure Monitor, the Sanofi iBGStar Blood Glucose Monitor, and the AliveCor Mobile ECG affected health care resource utilization measured by both health insurance claims and hospital visits. In their study, participants were split into control and intervention arms where those in the intervention arms utilized one of the 3 aforementioned technologies based on their health care needs. No difference between groups was observed for office visits (*P*=.46), inpatient stay (*P*=.82), emergency room visits (*P*=.06), or pharmacy claims (*P*=.60). Furthermore, no difference in self-efficacy change was observed between control and intervention group (*P*=0.85), and no difference in filed insurance claims between the 2 groups was observed (*P*=0.62) [46].

While future studies may examine whether mHealth technologies, such as the Ostom-i alert sensor, influence cost of care or duration of hospital stay, the purpose of the present study is to examine the extent to which the Ostom-i alert sensor affects the health-related quality of life of its users. To our knowledge, no such studies have been conducted, making this a unique undertaking.

## Abbreviations

BP: bodily pain
CoH-QOL-Ostomy: City of Hope Quality of Life Questionnaire for a Patient with an Ostomy
DMC: data monitoring committee
GH: general health perceptions
MH: mental health
OAS-SF2: Ostomy Adjustment Scale Short Form 2
PF: physical functioning
RE: role limitations due to emotional problems
REDCap: Research Electronic Data Capture
RP: role limitations due to physical health problems
SF: social functioning
SF-36: Medical Outcomes Study 36-item short form health survey
VT: vitality
